# Cardiorespiratory fitness in adolescents and young adults with Klinefelter syndrome – a pilot study

**DOI:** 10.3389/fendo.2023.1106118

**Published:** 2023-01-30

**Authors:** Julia Spiekermann, Kathrin Sinningen, Beatrice Hanusch, Michaela Kleber, Michael M. Schündeln, Cordula Kiewert, Heide Siggelkow, Jakob Höppner, Corinna Grasemann

**Affiliations:** ^1^ University Hospital of Pediatrics and Adolescent Medicine, St. Josef-Hospital, Ruhr-University Bochum, Bochum, Germany; ^2^ Center for Rare Diseases Ruhr (CeSER) , Ruhr-University Bochum and Witten/Herdecke University, Witten-Herdecke, Germany; ^3^ Division of Pediatric Hematology and Oncology, Department of Pediatrics III, University Hospital Essen, University of Duisburg-Essen, Essen, Germany; ^4^ Division of Pediatric Endocrinology, Department of Pediatrics II, University Hospital Essen, University of Duisburg-Essen, Essen, Germany; ^5^ Clinic of Gastroenterology, Gastrointestinal Oncology and Endocrinology, University Medical Center Goettingen, Goettingen, Germany and MVZ Endokrinologikum Goettingen, Goettingen, Germany

**Keywords:** Klinefelter syndrome, XXY, adolescence, children, cardiovascular disease, chronotropic insufficiency, physical activity

## Abstract

**Background:**

Klinefelter syndrome (KS) may be associated with a wide spectrum of phenotypic changes including endocrine, metabolic, cognitive, psychiatric and cardiorespiratory pathologies in adults. However, in adolescence the clinical phenotype of KS is not well described, especially regarding physical fitness. The present study reports on cardiorespiratory function in adolescents and young adults with KS.

**Methods:**

Adolescents and young adults with KS were recruited in a cross-sectional pilot study. Biochemical parameters of fitness including hormonal status, a body impedance analysis, the grip strength, the amount of physical activity at home for 5 days *via* trackbands and anamnestic parameters were assessed. In addition, participants underwent an incremental symptom-limited cardiopulmonary exercise test (CPET) on a bicycle ergometer.

**Results:**

Nineteen participants with KS aged 15.90 ± 4.12 years (range: 9.00 - 25.00) participated in the study. Pubertal status was Tanner 1 (n = 2), Tanner 2 - 4 (n = 7) and Tanner 5 (n = 10). Seven participants received testosterone replacement therapy. Mean BMI z-score was 0.45 ± 1.36 and mean fat mass was 22.93% ± 9.09. Grip strength was age-appropriate or above normal. 18 participants underwent CPET with subnormal results for maximum heart rate (z-score -2.84 ± 2.04); maximum workload (Watt_max_; z score -1.28 ± 1.15) and maximum oxygen uptake per minute (z- score -2.25 ± 2.46). Eight participants (42.1%) met the criteria for chronotropic insufficiency (CI). Data from track-bands showed sedentary behavior for 81.15% ± 6.72 of the wear time.

**Conclusion:**

A substantial impairment of cardiopulmonary function can be detected in this group of boys to young adults with KS, including chronotropic insufficiency in 40%. The track-band data suggest a predominantly sedentary lifestyle, despite normal muscular strength as assessed *via* grip strength. Future studies need to investigate the cardiorespiratory system and its adaption to physical stress in a larger cohort and in more detail. It is feasible that the observed impairments contribute to the avoidance of sports in individuals with KS and may contribute to the development of obesity and the unfavorable metabolic phenotype.

## 1 Introduction

Klinefelter syndrome (KS) is genetically determined by extra (and supra-numeric) X chromosome(s) resulting in the karyotype 47, XXY and its variants (1). The phenotypic presentation of individuals with KS is highly variable resulting in significant under-recognition of the syndrome despite a genetic prevalence of 0.1 - 0.2% (1–3). In childhood, there are few clinical signs which point to the presence of an additional X-chromosome in boys, except for a tall stature and difficulties in the school setting. However, beginning in mid- to late puberty a decline in testicular function can be observed in almost all individuals and the progressive destruction of testicular tissue results in hypergonadotropic hypogonadism with small and firm testes, gynecomastia and azoospermia (1). In addition, a broad spectrum of phenotypic and clinical changes of largely unknown etiology may emerge, with impairments of variable degrees in endocrine, metabolic, cognitive, psychological, cardiovascular and bone health.

Accordingly, registry-based studies from Denmark and UK demonstrate an elevated morbidity and reduced life expectancy in adults with KS (4–7). Changes of the metabolic risk profile in men with KS and an increased prevalence of type 2 diabetes likely contribute to this increased morbidity and mortality. Men with KS show a fivefold increased prevalence of metabolic syndrome compared to age-matched controls (8). Metabolic abnormalities can be detected early and even before the detectable onset of gonadal failure, children and adolescents with KS may develop an increased percentage of body fat and centrally located obesity, which may aggravate with older age (9–12). Bardsley et al. reported that in approximately 10% of prepubertal boys with KS a metabolic syndrome was detectable and insulin resistance was present in more than 24% (10).

These metabolic changes in turn are risk factors for cardiovascular disease and have been observed in adult individuals with KS (6, 13). For example, several studies have demonstrated dyslipidemia, diabetes mellitus (DM), abnormalities in biomarkers of cardiovascular disease and increased carotid intima media thickness as well as a reduction in systolic and diastolic function in adults with KS (7, 14).

In their study of 69 adults with KS, Pasquali et al. hypothesized that chronotropic insufficiency (CI) could be one of the causes of the observed cardiovascular abnormalities (14). CI refers to the inability of the heart to increase its rate adequately during physical stress. Indeed, adults with KS showed reduced cardiopulmonary exercise capacity (13) and Bojesen et al. demonstrated reduced peak oxygen uptake (VO2 max) in 70 adults with KS, with no difference between men with and without substitution of testosterone (5).

The emerging picture of multifactorial health impairments affecting multiple organ systems in adults with KS gives rise to the question of the overall health in children, adolescents, and young adults with KS. Here we report on physical fitness and cardiorespiratory function in a well-characterized cohort of children and adolescents with KS.

## 2 Materials and methods

### 2.1 Cohort

Between April 2021 and May 2022, 19 individuals with KS between 9 and 25 years were recruited into the KliBONE Study (DRKS Registration No.: DRKS00024870) at the Department for Pediatrics of the Ruhr-University Bochum, Bochum, Germany. Patients were recruited during the annual screening visits. In addition, participants were recruited *via* the national patient groups for XXY (15, 16). Inclusion criteria were a confirmed diagnosis of Klinefelter syndrome, age between 9 and 25 years and signed informed consent from the participants and/or from their legal guardians if applicable. The study protocol was approved by the Ruhr-University Bochum Ethics Committee (#21-7164). The study was conducted in accordance with the principles of the Declaration of Helsinki.

### 2.2 Clinical parameters

Clinical and anamnestic parameters were assessed as following: anamnestic parameters included age, age at diagnosis, birth weight and birth length, height of both parents, current medication, tobacco and alcohol use, medical history and detailed information on start, frequency and dosing of testosterone treatment if applicable. A physical exam was performed assessing patient height, weight and pubertal stage according to Tanner stage. Standing height was measured using a wall-mounted stadiometer (Ulmer Stadiometer, Busse Design, Elchingen, Germany) to the nearest mm. Weight was recorded to the nearest 0.1 kg using a digital scale (Seca, Hamburg, Germany). BMI was calculated from these data using the formula weight (kg)/(height in m)^2^. Z-scores and percentiles were calculated based on the Centers for Disease Control and Prevention (CDC) data (17). An experienced pediatric endocrinologist assessed the pubertal development according to the Tanner stages and the testicular volume was assessed using a Prader orchidometer. Clinical and anamnestic parameters were assessed as previously described in detail (18, 19). Each participant also underwent a body impedance measurement using the TANITA Body composition analyzer (Model DC-360, TANITA Europe B. V., Amsterdam, the Netherlands).

### 2.3 Laboratory tests

Biochemical parameters were assessed in serum or plasma samples as part of the routine diagnostic laboratory workup in the central laboratory of the St. Josef-Hospital Bochum and at the MVZ Dr Eberhard & Partner Dortmund, Germany. Blood was drawn before 10 am in all participants and send for analyses within two hours. Additional serum, plasma and urine aliquots were stored at -80°C until further analysis. The following parameters were included into the analysis of this study: Hemoglobin (Hb, g/dl), luteinising hormone (LH, mIU/ml), follicle stimulating hormone (FSH, mIU/ml), and testosterone (ng/ml). In addition, parameters of thyroid function, including thyroid-stimulating hormone (TSH, uIE/ml), free triiodothyronine (fT3, pg/ml) and thyroxine (fT4, ng/dl) were determined.

### 2.4 Grip strength

Grip strength is considered a reliable indicator of a person’s muscular strength (20). Hand grip strength (HGS) was measured according to Dodds et al. using a Jamar handgrip dynamometer (Promedics, Blackburn, UK). The measurements were performed as following: In a sitting position the proband rested the arm at a right angle on the leg (21). As recommended by Richards et al., the forearm was positioned in supination (22). The proband pressed the handle of the dynamometer with maximum force. A total of three measurements of each hand (right and left) were taken; the respective maximum value was recorded in kilograms. Individual z-scores were calculated using the grip strength z-score calculator based on the reported values by Dodds et al. (21, 23).

### 2.5 Cardiopulmonary exercise test

18 patients performed a symptom-limited cardiopulmonary exercise test (CPET) on a bicycle ergometer (VIAsprint^®^ ergoselect 100 ergometer, ergoline GmbH, Bitz, Germany). The test implementation was adapted to the methods previously described by Pasquali et al. (14) Pedal length was adjusted according to the recommendations of the German society for pediatric cardiology (24, 25). As recommended for children and adolescents, a weight-based protocol was used with a starting load of 1.0 W/kg body weight and an increase of 0.5 W/kg load every two minutes (24, 26). The test was continued until limiting symptoms for exercise termination occurred, according to criteria previously described (27). For overweight children and adolescents, the workload was calculated corresponding to their height to avoid overloading (26). Respiratory gas exchange measurements were obtained breath by breath by a commercially available system (Vyntus^®^ CPX, Vyaire medical Inc., Mettawa, IL-USA). Peak oxygen uptake (VO2) was recorded as the mean value of VO2 during the last 20 s of the test. In addition, the respiratory volume per minute, the breathing rate per minute and the heart rate per minute were measured. All parameters were recorded at the start of the test (resting conditions) and continuously during the test. When the maximum workload was reached, the system was immediately resetted to the individual starting conditions and measurements were obtained for another two minutes. As recommended by the German society for pediatric cardiology, individual z-scores for participants ages 6 – 18 were calculated based on the reported data by Klemt et al. (28, 29) For participants >18 years, the reference values reported by Gläser et al. from the SHIP-Study (30) were used.

### 2.6 Chronotropic insufficiency

Chronotropic insufficiency (CI) was defined if one of the following conditions applied: 1) Maximum Heart rate (HR) below 85% of age-appropriate maximum HR or 2) HR increase below 80% of adjusted (percent) HR reserve, determined from the change in HR from the rest to peak exercise divided by the difference of the resting HR and age-predicted maximal HR $\left(\frac{HR_{max}-\;HR_{rest}}{Age\;predicted\;HR_{max}-\;HR_{rest}}\right)$ (14, 31). Age-predicted maximal HR was calculated according to the formular previously described by *Mahon et al.* for children and adolescent (208 – 0.7 x Age) (32).

Heart recovery rate (HRR) was assessed as the decrease in HR from peak exercise to HR after 2 min of “active” cycling with the start load (33).

### 2.7 Accelerometer

Participants were asked to wear an accelerometer (wGT3X-BT; ActiGraph LLC, Pensacola, Florida) for seven consecutive days. The accelerometers were equipped with a tri-axial acceleration sensor. Data sets from accelerometers were considered for analysis only if a minimum wear (and recording) time of eight hours on four weekdays and one weekend day was achieved, consistent with the criteria for inclusion in the International Children’s Accelerometry Database (ICAD) (34). Each ActiGraph activity monitor was initialized using a standardized procedure before use by the participant. The monitors used the latest firmware (v1.9.2. for wGT3X-BT and v3.2.1 for GT3X+), a unique output filename, and a sampling frequency of 30 Hz.

The device was set up to start the measurement at 12:00 AM the day after the examination and to stop the measurement at midnight after seven days of recording (35). The device was placed laterally on top of the right anterior superior iliac spine with the closure on top, then secured with an elastic belt (35). Participants were asked to complete a non-wear time protocol. The wear time values from the non-wear time protocols were compared with the calculated values of different non-wear time algorithms by Choi et al., using a 90-minute window ( ± 30 minutes) for capturing non-wear time (36). The data were downloaded from the devices as gt3x files using ActiLife Version 6.13.3 software (ActiGraph). Data sets with less than 4 + 1 days of wear time were excluded from analysis. As recommended by Edwardson et al. data were downloaded in epoch lengths of one second (37).

According to previously published algorithms, we categorized physical activity (PA) into sedentary, light, moderate and vigorous PA. The classification into these groups were applied according to Evenson et al. (38).

### 2.8 Statistical analysis

Statistical analysis was performed using Jamovi 2.3 version 1.6 for Mac (The jamovi project [2021]). Retrieved from jamovi (version 1.6) (39). Data are presented as mean ± standard deviation (SD) of mean. Data were tested for normal distribution using the Shapiro-Wilk test. Mann-Whitney-U test (non-parametric) or the unpaired *t*-test (parametric) were performed for continuously distributed variables for comparison between two groups. For categorical variables the Chi-Square test or Fisher’s exact test was performed. A p-value of < 0.05 was considered statistically significant. Due to the exploratory nature of the study no adjustment for multiple testing was applied.

## 3 Results

### 3.1 Participants

In total, 19 participants with KS were enrolled in this study. The age at inclusion ranged between 9 and 25 years, the mean age at enrolment was 15.90 (± 4.12, range 9 - 25) years. KS was diagnosed at a mean age of 6.50 (± 6.59) years in the cohort. Mean height z-score was 1.53 (± 1.07, range -0.2 – 4.2) and the mean BMI z-score was 0.45 (± 1.36). The mean average arm span did not differ from the mean height (179.9 cm ± 15.62 vs 177.81 cm ± 13.04 for height). Hemoglobin was in the normal range in all participants. For detailed clinical and laboratory information refer to **Table 1**.

**Table 1:** Clinical and laboratory characteristic of 19 participants with KS.

Clinical parameters	
Age at visit (years)	15.90 ± 4.12 (9.00 – 25.00)
Age at diagnosis (years)	6.50 ± 6.59 (0.00 – 17.00)
Height (z-score)	1.53 ± 1.07 (-0.20 – 4.20)
Weight (z-score)	1.18 ± 0.93 (-0.37 – 2.68)
BMI (z-score)	0.45 ± 1.36 (-2.00 – 2.50)
Fat mass (%, Impedance Scale)	22.93 ± 9.09 (9.20 – 38.30)
Selected laboratory parameters [reference values]	
Hemoglobin (g/dl) [14.0 – 18.0]	14.15 ± 1.14 (12.10 – 16.00)
TSH (uIE/ml) [0.51 – 4.30]	1.96 ± 1.03 (0.57 – 4.33)
fT3 (pg/ml) [2.56 – 5.01]	3.65 ± 0.64 (2.21 – 4.53)
fT4 (ng/dl) [0.90 – 2.10]	1.13 ± 0.20 (0.87 – 1.59)

Continuous data are shown as mean ± standard deviation, range (min – max). Reference values are provided in square brackets.

BMI, body mass index; fT3, free triiodothyronine; fT4, thyroxine; TSH, thyroid-stimulating hormone.

With respect to pubertal development the distribution of Tanners stages (PH and G-stages) was as following: Tanner 1: n = 2; Tanner 2: n = 1; Tanner 3: n = 2, Tanner 4: n = 4 and Tanner 5: n = 10. Of the participants with adult pubertal status, 7 had developed hypergonadotropic hypogonadism in the past and were on regular testosterone replacement therapy (testosterone undecanoate 250 mg/3-4 weekly (n = n=3); testosterone undecanoate 1000 mg/3montly (n = 1), testosterone gel 25 - 50 mg/transdermal daily (n = 3)). For distribution of testosterone, LH and FSH levels in the cohort refer to **Supplementary Figure 1**.

Concomitant medication with potential relevance to cardiorespiratory function in this group included Celecoxibe (n =1), Methylphenidate (n = 4), Pregabaline (n = 1), Risperidone (n = 1), Salbutamol (n = 1).

### 3.2 Grip strength and Track-band Analysis

**Figure 1:**
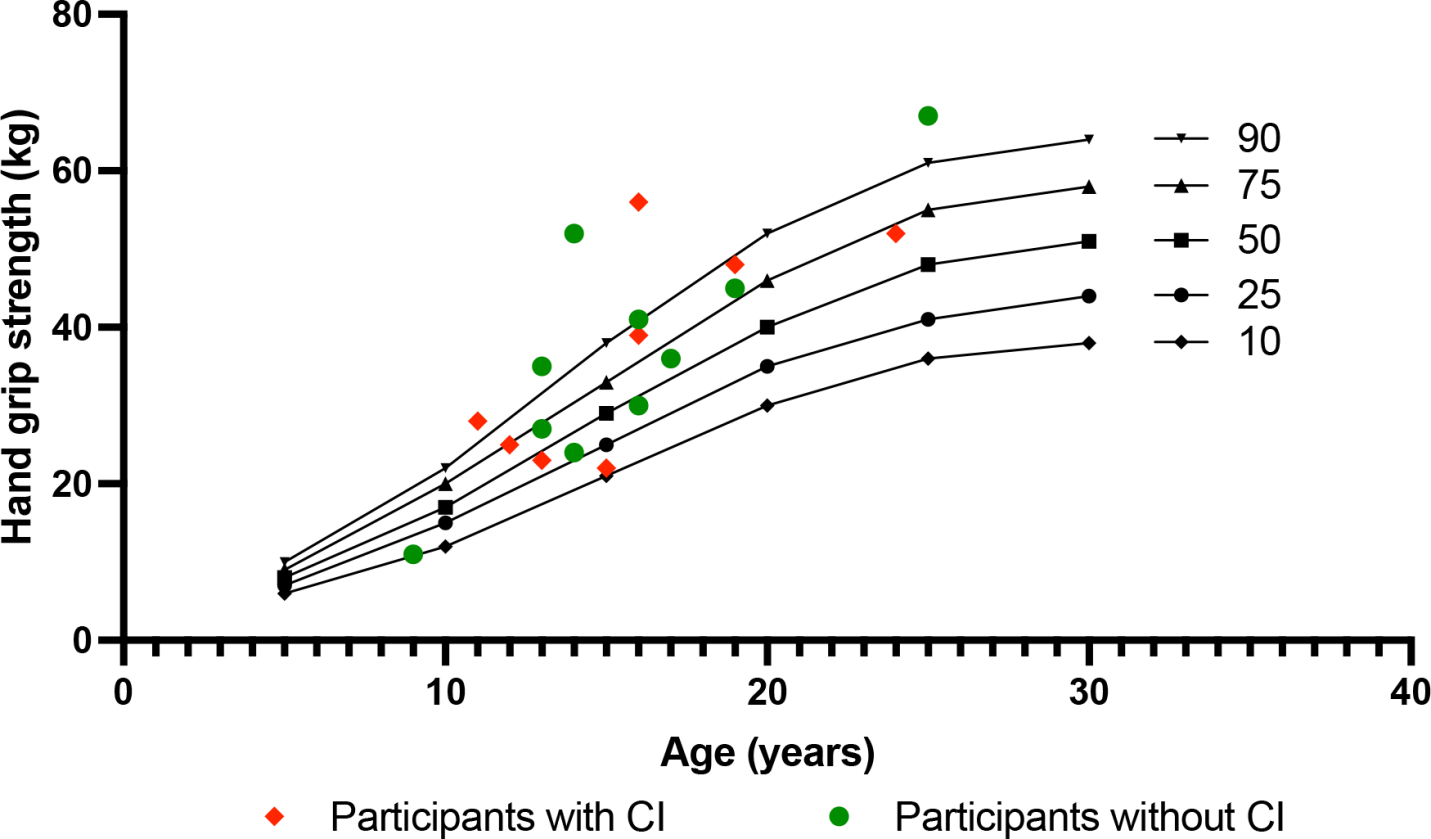
Hand grip strength. Results of hand grip strength measurement in participants with KS (red squares and green circles) and age-appropriate percentiles according to Dodds et al. (21) Presence of chronotropic insufficiency (CI) in participants is indicated by color (red squares = with CI; green circles = without CI).

Grip strength was measured in 18 participants. The mean maximum grip strength was 36.72 kg (± 14.47; range 9 - 67), which corresponds to a mean z-score of 0.84 ± 1.32. Data and Distribution are displayed in **Table 2** and **Figure 1**.

**Table 2:** Grip strength and Track-band results of 18 participants with KS.

Grip strength	
Maximum grip strength (kg)	36.72 ± 14.47 (11.00 – 67.00)
Maximum grip strength (z-score)	0.84 ± 1.32 (-1.26 – 3.74)
Track-band	
Average daily wear time (hours)	11.21 ± 2.81
Sedentary breaks (in % wear time)	81.15 ± 6.72 (67.56 – 92.45)
Light Physical Activity (in % wear time)	11.88 ± 3.74 (5.41 – 20.64)
Moderate Physical Activity (in % wear time)	3.40 ± 1.46 (1.48 – 5.94)
Vigorous Physical Activity (in % wear time)	3.56 ± 4.23 (0.65 – 19.11)
Percent of MVPA	6.96 ± 5.03 (2.13 – 23.60)

Continuous data are shown as mean ± standard deviation, range (min – max) is given in brackets. MVPA, moderate-to-vigorous physical activity; PA, physical activity.

Physical activity was recorded *via* the ActiGraph Track-band in 18 participants for a mean of 6.33 (± 1.28) days with a mean daily wearing time of 11.21 (± 2.17) hours. On average, data showed sedentary behavior during 81.15% (± 6.72) of the wear time and light physical activity for 12.06% (± 3.77). Vigorous physical activity was rarely performed, with an average of 2.65% (± 1.74) of the wear time (**Table 2**).

### 3.3 Cardiopulmonary exercise test (CPET)

Cardiopulmonary exercise testing (CPET) was performed in 18 participants (age range 11 – 25 years). One study participant was excluded from the exercise test for medical reasons. **Table 3** shows the results of the test under resting conditions and at maximum workload.

**Table 3:** Cardiopulmonary Exercise Test results of 18 participants with KS.

Parameters under resting conditions	
Heart rate (bpm)	82.22 ± 12.31 (60.00 – 102.00)
Systolic blood pressure (mmHg)	114.20 ± 15.49 (91.00 – 139.00)
Diastolic blood pressure (mmHg)	65.33 ± 13.68 (45.00 – 101.00)
Respiratory volume (l/min)	13.77 ± 3.77 (9.00 – 20.00)
Breathing rate (/min)	21.24 ± 2.90 (17.00 – 27.00)
Oxygen uptake ml/min)	396.00 ± 95.19 (215.00 – 553.00)
Starting workload (W)	67.78 ± 9.27 (50.00 – 85.00)
Parameters at maximum load	
Workload (W)	160.33 ± 49.02 (90.00 – 280.00)
Workload (z-score)	-1.82 ± 1.40 (-4.40 – 0.22)
Respiratory volume (l/min)	68.12 ± 19.38 (45.00 – 101.00)
Respiratory volume (z-score)	-2.05 ± 1.38 (-4.08 – 0.41)
Breathing rate (/min)	42.31 ± 9.52 (29.70 – 72.30)
Breathing rate (z-score)	-0.69 ± 1.17 (-2.44 – 1.37)
Oxygen uptake (ml/min)	2078.12 ± 573.39 (1380.00 – 3129.00)
Oxygen uptake (z-score)	-2.76 ± 2.27 (-6.88 – 0.22)
Heart rate (bpm)	168.33 ± 18.31 (126.00 – 194.00)
Heart rate (z-score)	-2.84 ± 2.04 (-7.97 – -0.49)
Heart rate at maximum load / calculated maximum heart rate	0.86 ± 0.09 (0.65 – 0.99)
Heart rate reserve (%)	76.90 ± 14.70 (42.60 – 97.20)
Examination time (min)	7.58 ± 2.56 (5.00 – 13.50)

Continuous data are shown as mean ± standard deviation, range (min – max) is given in brackets. Heart rate reserve (HRR) was calculated as the change in HR from the rest to peak exercise divided by the difference of the resting HR and age-predicted maximal HR. HR, Heart rate; V’E, Respiratory volume; BR, Breathing rate; V’O2, Oxygen uptake; HRR, Heart rate reserve.

**Figure 2:**
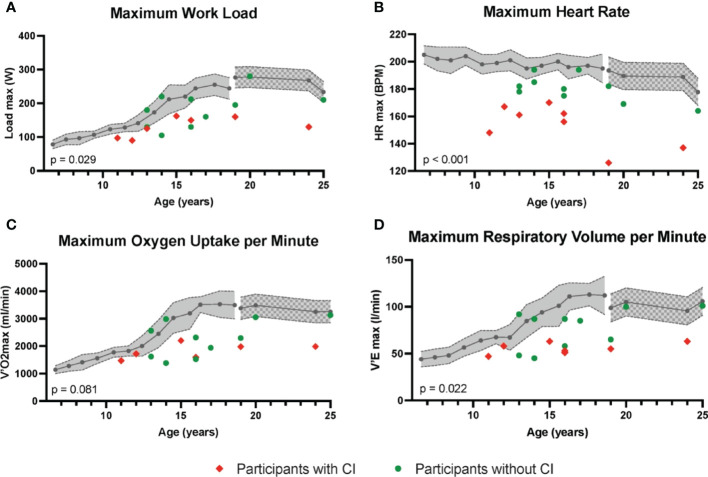
Cardiopulmonary exercise test (CPET). Results of CPET in participants with KS (red squares and green circles) expressed as **(A)** maximum workload achieved on the ergometer in Watt, **(B)** the maximum heart rate in BPM, **(C)** the maximum oxygen uptake per minute [ml/min] and **(D)** the respiratory minute volume [l/min]. The grey area indicates the age-appropriate mean and standard deviation according to the results of Klemt et al. (28) for children and adolescents aged 6 to 18 years; the gray patterned area indicates the age-appropriate mean and standard deviation according to the results of Gläser et al. for adults (30). Participants with chronotropic insufficiency (CI) are shown as red squares; participants without chronotropic insufficiency as green circles. HR, Heart rate; V’O2 max, Maximum oxygen uptake; V’E max, Maximum respiratory volume; CI, Chronotropic insufficiency.

Participants reached a maximum workload with a mean z-score of -1.82 (± 1.40). Also, the breathing rate per minute (42.31 ± 9.52/min), the respiratory volume per minute (V’E 68.12 ± 19.38 l/min) and the maximum oxygen uptake per minute (V’O2 2078.12 ± 573.40 ml/min) were reduced in participants with KS (**Figure 2**).

The heart rate increased to an average of 168.33 (± 18.31, mean z-score of -2.84 (± 2.04) beats per minute (bpm) at maximum load, this corresponds to 86% (± 9) of the calculated age-appropriate maximum heart rate. With regard to the performance in the CPET, no significant differences were found between participants with testosterone replacement therapy and those without testosterone replacement therapy. However, a positive correlation was found between serum testosterone levels and maximum power on the bicycle ergometer (r = 0.55, p = 0.02; **Supplementary Table 3**).

### 3.4 Chronotropic insufficiency

Chronotropic Insufficiency (CI) was detected in 8 of 18 adolescent participants (44.4%) (**Table 4**).

**Table 4:** Comparative analysis between participants without and with Chronotropic insufficiency (CI).

Clinical Item	Without CI (n = 10)	With CI (n = 8)	P Value
Patient’s characteristics			
Age at visit (years)	16.70 ± 3.77	15.75 ± 4.20	0.620
BMI (z-score)	0.80 ± 1.20	-0.14 ± 1.44	0.151
Testosterone Therapy	4 (40%)	3 (38%)	1.000
Laboratory values			
Hemoglobin (g/dl)	14.22 ± 1.25	14.01 ± 1.14	0.820
Grip strength			
Maximal grip strength (kg)	39.67 ± 13.53	36.63 ± 13.92	0.655
Maximal grip strength (z-score)	1.09 ± 1.29	0.88 ± 1.27	0.739
Track-band			
Percent of sedentary breaks	83.85 ± 6.20	80.84 ± 4.76	0.306
Percent of MVPA	4.95 ± 2.47	6.87 ± 3.25	0.200
Cardiopulmonary Exercise Test			
Heart rate at rest (bpm)	88.90 ± 8.92	80.88 ± 11.81	0.120
Breathing rate at rest (/min)	21.07 ± 3.36	21.47 ± 2.34	0.789
Respiratory volume at rest (l/min)	13.80 ± 3.79	13.71 ± 4.03	0.965
Oxygen uptake at rest (ml/min)	411.30 ± 74.47	374.14 ± 122.06	0.446
Workload at start (W)	69.50 ± 9.56	65.63 ± 9.04	0.394
Maximum workload (W)	182.20 ± 52.41	133.00 ± 27.69	0.029
Maximum breathing rate (/min)	43.72 ± 11.66	40.29 ± 5.46	0.482
Maximum respiratory volume (l/min)	76.80 ± 20.99	55.71 ± 6.02	0.022
Maximum oxygen uptake (ml/min)	2280.30 ± 652.84	1789.29 ± 268.32	0.081
Maximum heart rate (bpm)	180.30 ± 9.60	153.38 ± 15.34	< 0.001
Heart rate at maximum load / calculated maximum heart rate (%)	91.80 ± 4.20	77.80 ± 7.20	< 0.001
Heart rate reserve (%)	87.90 ± 5.90	63.10 ± 9.60	< 0.001
Examination time (min)	8.31 ± 2.99	6.66 ± 1.64	0.182
Heart recovery rate (HRR)	39.11 ± 16.77	45.86 ± 11.88	0.383

When participants without CI (n = 10) were compared to participants with CI (n = 8), there were no significant differences between the two groups regarding height, weight, pubertal status or testosterone therapy. Likewise, there were no differences regarding grip strength. However, performance in CPET differed between the subgroups: Participants with CI achieved significantly less exertion on the bicycle ergometer (133.00 Watt [W] ± 27.69 vs. 182.20 W ± 52.41 in participants without CI; p = 0.029), showed a significantly reduced respiratory volume per minute (55.71 l/min ± 6.02 vs. 76.80 l/min ± 20.99; p = 0.022) and a trend towards a reduced oxygen uptake per minute (1789.29 ml/min ± 268.32 vs. 2280.30 ml/min ± 652.84; p = 0.081) (**Figure 2**). No differences in respiratory rate during maximum exercise (40.29/min ± 5.46 vs. 43.72/min ± 11.66; p = 0.482) and the heart recovery rate (HRR) after two minutes (45.86 ± 11.88 vs. 39.11 ± 16.77; p = 0.383) were observed.

## 4 Discussion

With this study we report data on physical activity and fitness in adolescent and young adult men with KS and show a striking impairment in cardiorespiratory fitness in most of the participants of the study.

While it is known that children and adolescents with KS tend to avoid competitive sports even before the development of hypergonadotropic hypogonadism, the extend of the observed impairment of physical fitness in the bicycle ergometric test and the amount of sedentary behavior in the home environment in this group was unexpected.

In the ergometry testing a reduced maximum performance as well as significant limitations in respiratory function regarding respiratory minute volume and maximum oxygen uptake were observed. These data are in line with data reported by Bojesen et al. that show changes in V’O2 in adults with KS (5).

Chronotropic insufficiency may contribute to the results of the ergometry testing. The term “chronotropic insufficiency” describes the inability to adequately increase the heart rate during physical stress. In this cohort, the maximum heart rate (at maximum performance) was reduced and corresponded to a mean z-score of -2.84 (± 2.04) of the calculated age-appropriate values, thereby matching the description of a chronotropic insufficiency in almost half of the cases. Previously, Pasquali et al. conducted spiroergometric exercise testing in 48 adults with KS (mean 30 ± 3 years) and described CI in 55% of the patients. CI is a common finding in patients with cardiovascular disease and the resulting inability to compensate for prolonged exercise by increasing the heart rate is an independent predictor of serious cardiovascular events and all-cause mortality in asymptomatic populations. Individuals tend to avoid exercise and may experience a reduced quality of life (31, 40, 41).

The heart rate is primarily regulated *via* the autonomic nervous system, including the sympathetic and parasympathetic nervous systems. The positive chronotropic effect is mediated *via* the activation of the ß1 receptors by means of adrenaline and noradrenaline, which in turn causes an increase in the cAMP concentration in sinus node cells (42). It has been hypothesized, that the response of the autonomic nervous system to physical stress may be impaired in a subgroup of individuals with KS. There are reports which support this hypothesis, e.g., Hainstock et al. reported on postural orthostatic tachycardia syndrome (POTS) in a teenager with KS (43) and indeed sympathetic denervation of the lower extremities is a leading theory as cause for POTS (44). Moreover, there are several case reports in the literature of nervous system involvement in KS (45–48). Potential causes of neuropathy in KS may be testosterone deficiency, diabetes mellitus and thyroid dysfunction (8, 10, 49). However, in this cohort of young individuals with KS none had developed diabetes mellitus or thyroid dysfunction and testosterone deficiency had not yet developed or developed quite recently and was adequately treated.

The presence of CI in the reported cohort was associated with poorer performance in the exercise test, including lower maximum pedaling performance, a trend for shorter exercise time on CPET and impaired respiratory parameters. E.g., the maximum respiratory volume per minute of the CI group was significantly reduced in contrast to boys without CI. Respiratory minute volume is defined as the volume of air inhaled or exhaled per minute. A reduced respiratory minute volume can therefore be caused by both disturbed regulation of the respiratory rate and a reduced volume capacity. In the described cohort, the maximum respiratory rate did not differ between participants with CI and without CI. The cause for the differences in respiratory minute volume (V`E in ml/min) and oxygen uptake per minute (V’O2 in ml/min) must therefore lie in the respiratory volume. Indeed recently, Zhao et al. observed an association of pulmonary disease including chronic obstructive pulmonary disease with KS (50). In order to exclude a pulmonary etiology with certainty, a lung function test should be carried out in future examinations.

In addition to an impaired function of the cardiorespiratory system, muscular exhaustion could be an explanation for the early termination in the ergometry testing in this group. To investigate muscular strength in the participants maximum hand grip strength was assessed, which is considered a reliable indicator of total muscle strength (20). Interestingly, maximum hand grip strength of the participants was within the normal range or even slightly above the norm which suggests that muscle strength itself is not impaired.

In KS low or subnormal testosterone levels may play a role for physical strength even in treated or pubertal participants. However, the influence of low testosterone levels on the cardiovascular system is unclear. Some data associate low testosterone levels with increased cardiovascular mortality (51) and testosterone is considered a protective factor regarding cardiovascular risk factors. Caminiti et al. showed a positive influence of testosterone replacement therapy on muscle strength and improved performance in patients with heart failure (52). In this cohort, participants showed either adequate (= age and pubertal status appropriate) testosterone levels or were on a regular testosterone replacement therapy. No significant differences in testosterone levels were found regarding testosterone levels between the participants with or without CI.

The results of the CPET are further supported by the data from the trackbands. In this study, KS participants spent 82% of the wear time with sedentary activities and only 6% with moderate-to-vigorous physical activity (MVPA), corresponding to 42.83 ± 5.03 minutes. This is less engagement in physical activity than data from the nation-wide study on healthy children and adolescents, the KiGGs study of the Robert-Koch Institute, indicates in healthy controls. In the KiGGs study, 6–17-year-olds spent 69% of the time in sedentary activities and about 6.5% in MVPA (53). According to the current WHO guideline, children and adolescents aged 5-17 years are recommended to engage in at least 60 minutes per day into moderate-vigorous physical activity (54). The children and adolescents with KS of the present study achieve only one tenth of these recommendations.

It is noteworthy, that only few individuals of this cohort were engaged in organized sports with regular physical training. Two of the participants achieved an age-appropriate performance in the bicycle ergometry testing. However, it remains unclear whether these individuals were able to participate in organized sports and regular fitness training because of a better pre-existing function of the cardiorespiratory system, or whether the regular training had corrected a potentially impaired system.

The results of this study raise multiple questions regarding the reduced physical performance in the tested individuals. The limited cardiorespiratory function and even the presence of a CI could be a pre-existing condition which inhibits boys and adolescents with KS to engage in physical activity or even to engage in a more active lifestyle in the domestic setting. The development of comorbidities, e.g., obesity, metabolic syndrome, diabetes mellitus and even the elevated risk for thrombosis (55) may be secondary developments of a sedentary lifestyle that may call for specific interventions and programs.

Future studies are needed to confirm the results of this study in larger study groups. Furthermore, studies on the function of the autonomic nervous system and the pulmonary function in individuals with KS are necessary to further investigate this phenomenon. An international registry would help to understand the development of the different phenotypic aspects of KS over the lifetime.

### 4.1 Study limitations

Aside from cardiorespiratory and muscular malfunctions, a lack of motivation to challenge oneself could lead to subnormal results in a bicycle ergometry test. While we cannot fully exclude motivational aspects as a confounding parameter, a major strength of this study is the standardization of procedures. The bicycle ergometry testing was performed following standardized protocols by a single investigator, who encouraged participants to maximum performance individually. Previously published and recommended protocols were used. Nevertheless, no control group was enrolled, but published reference values were used. For this reason, we restricted the statistical testing to differences within the KS cohort.

## 5 Conclusion

With the present study, we report that children, adolescents, and young adults with KS show reduced cardiopulmonary performance without evidence for a muscular cause for this finding. Criteria for chronotropic insufficiency were fulfilled in 44% of the participants and point towards a possible role of the autonomous nervous system for the impaired function. Should this finding be confirmed by others it may explain the avoidance of physical activity and sports in many individuals with KS and may, at least in part, be causative for the development of obesity and comorbidities which result in increased morbidity and mortality in KS over time.

## Data availability statement

The original contributions presented in the study are included in the article/**Supplementary Material**. Further inquiries can be directed to the corresponding author.

## Ethics statement

The studies involving human participants were reviewed and approved by Ruhr-University Bochum Ethics Committee (#21-7164). Written informed consent to participate in this study was provided by the participants’ legal guardian/next of kin.

## Author contributions

Study design: CG, JH, and JS; Study conduct and recruitment: CG, JH, JS, BH, KS, CK, and HS; Data analysis and interpretation: JH, MS, JS, and CG; Drafting manuscript: JS, JH, and CG; Revising manuscript content: All authors. All authors contributed to the article and approved the submitted version.
