# Supplementary material for: Cardiorespiratory fitness in adolescents and young adults with Klinefelter syndrome – a pilot study
Source: Front Endocrinol (Lausanne). 2023 Jan 30;14:1106118. doi: 10.3389/fendo.2023.1106118 (PMC9922696; doi:10.3389/fendo.2023.1106118)
Supplement: Supplementary file 1 [file DataSheet_1.pdf]

Supplementary Material

Cardiorespiratory fitness in adolescents and young adults with Klinefelter Syndrome – a pilot study

Julia Spiekermann[1,2], Kathrin Sinningen[1], Beatrice Hanusch[1], Michaela Kleber[1], Michael M. Schündeln[3], Cordula Kiewert[4], Heide Siggelkow[5], Jakob Höppner[1,2,*], Corinna Grasemann[1,2,*]

Suppl. Table 1: Laboratory parameters of 19 participants

**Already determined parameters**	
**Hemoglobin** (g/dl)	14.15 ± 1.14
**TSH** (uIE/ml)	1.96 ± 1.03
**fT3** (pg/ml)	3.65 ± 0.64
**fT4** (ng/dl)	1.13 ± 0.20
**LH** (mlU/ml)	8.79 ± 9.38
**FSH** (mlU/ml)	17.48 ± 16.35
**Testosterone** (ng/ml)	3.50 ± 2.95
**Cortisol** (ug/dl)	
**25-OH-Vitamin D (ng/ml)**	

Continuous data are shown as mean and standard deviation (SD) categorical data as count and percentages.

LH - luteinising hormone; FSH - follicle stimulating hormone; TSH - thyreoid-stimulating hormone; fT3 - free triiodothyronine; fT4 - thyroxine,

Suppl. Table 2: Detailed information of 19 participants

Study ID	Age at visit (years)	Age at diagnosis (years)	Height (cm)	Armspan (cm)	BMI (kg/m^2)	Testosterone Replacement Therapy
1	14	0	172,4	171	24,6	/
2	25	17	178	n/A	38,7	250mg testosteronenantat i.m./3 weeks
3	13	0	166,2	165,5	18,2	/
4	16	0	188,4	187	17,9	250mg testosteronenantat i.m. /4 weeks
5	11	7	174,7	177	21,2	/
6	24	n/A	188,4	190	18,8	Method of therapy unknown
7	9	9	138,6	138	21,1	/
8	19	17	187	189	19,2	transdermal testosterone 50mg
9	15	0	181	n/A	19,4	/
10	13	12	177,4	n/A	18,2	/
11	16	13	192,4	194	22,7	transdermal testosterone
12	20	4	191,3	193	23,9	transdermal testosterone 50 mg
13	19	11	191	197	18	250mg testosteronenantat i.m. /4 weeks
14	12	1	158,2	159	26,1	/
15	14	0	180,8	185	18,4	/
16	13	0	176,8	180	24	/
17	16	n/A	183,8	194	23,9	/
18	16	n/A	172,4	175	26,8	/
19	17	13	179,5	185	31,5	/

ID – identification number; BMI – body mass index

Supp. Table 3: Correlation of Serum Hormones and CPET Performance Parameters

Serum Hormones	CPET Parameters	Pearson's r	p-Value
**Testosterone** (ng/ml)	Workload (z-score)	0.551	**0.022**
	Heart rate (z-score)	0.356	0.161
	Breathing rate (z-score)	0.461	0.072
	Respiratory volume (z-score)	0.466	0.069
	Oxygen uptake (z-score)	0.328	0.215
**FSH** (mlU/ml)	Workload (z-score)	-0.137	0.588
	Heart rate (z-score)	0.032	0.901
	Breathing rate (z-score)	-0.231	0.372
	Respiratory volume (z-score)	-0.108	0.680
	Oxygen uptake (z-score)	0.108	0.681
**LH** (mlU/ml)	Workload (z-score)	0.147	0.561
	Heart rate (z-score)	0.335	0.175
	Breathing rate (z-score)	-0.154	0.775
	Respiratory volume (z-score)	0.010	0.969
	Oxygen uptake (z-score)	-0.053	0.841

LH - luteinising hormone; FSH - follicle stimulating hormone

Supp. Figure 1

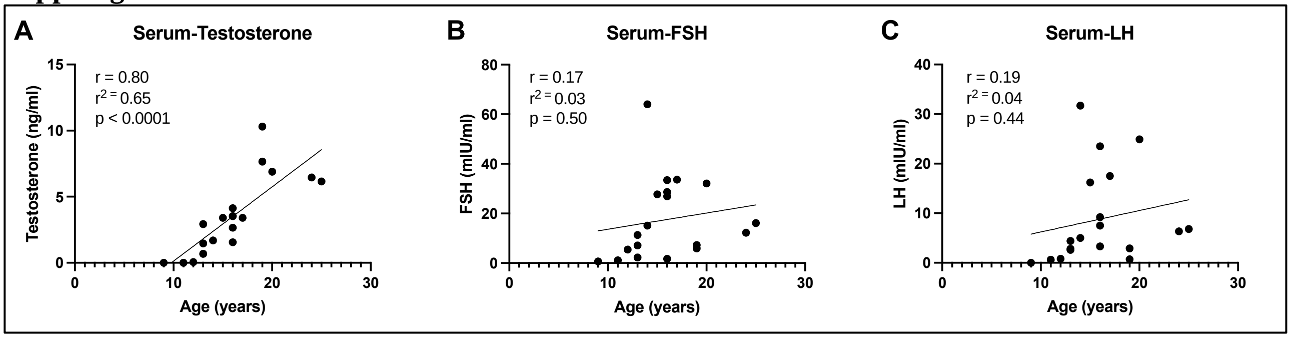

Fig. 1.: Serum Hormone Levels and age (in years) of the participants. A: Serum testosterone, B: Serum FSH, C: Serum-LH; FSH - follicle stimulating hormone; LH - Luteinizing hormone
